# Recombination across distant coronavirid species and genera is a rare event with distinct genomic features

**DOI:** 10.1128/jvi.01100-24

**Published:** 2024-11-19

**Authors:** Juan Patiño-Galindo, Adolfo García-Sastre, Jens H. Kuhn, Raul Rabadan, Gustavo Palacios

**Affiliations:** 1 Department of Microbiology, Icahn School of Medicine at Mount Sinai, New York, New York, USA; 2 Global Health Emerging Pathogens Institute, Icahn School of Medicine at Mount Sinai, New York, New York, USA; 3 Department of Medicine, Division of Infectious Diseases, Icahn School of Medicine at Mount Sinai, New York, New York, USA; 4 The Tisch Cancer Institute, Icahn School of Medicine at Mount Sinai, New York, New York, USA; 5 Department of Pathology, Molecular and Cell-Based Medicine, Icahn School of Medicine at Mount Sinai, New York, New York, USA; 6 The Icahn Genomics Institute, Icahn School of Medicine at Mount Sinai, New York, New York, USA; 7 Integrated Research Facility at Fort Detrick, National Institute of Allergy and Infectious Diseases, National Institutes of Health, Fort Detrick, Frederick, Maryland, USA; 8 Department of Systems Biology, Program for Mathematical Genomics, Columbia University, New York, New York, USA; Cornell University Baker Institute for Animal Health, Ithaca, New York, USA

**Keywords:** SARS-CoV-2, evolution, genetic recombination

## Abstract

Severe acute respiratory syndrome coronavirus 2 (SARS-CoV-2; family *Coronaviridae*, genus *Betacoronavirus*, subgenus *Sarbecovirus*) has caused millions of deaths, prompting a need for better understanding of coronavirid emergence and spillover to humans. As an evaluation of how some features of SARS-CoV-2, unique among sarbecoviruses, may have been acquired from related viruses, we conducted phylogenetic and recombination analyses to compare the frequency of recombination among coronavirids across vs within genera, subgenera, and species. Among known betacoronaviruses, we identified 199 (183 intraspecies, 16 interspecies, but no intersubgenera) recombination events. Phylogenetic analyses revealed that the ancestry of interspecies events was limited and less prone to affect 5′ regions of coronavirid genome open reading frame 1 (ORF1) than intraspecies events. On the contrary, interspecies events were significantly more prone to impact the 3′ end (ORF6–ORF8 and the nucleocapsid protein [*N*] ORF), suggesting the existence of region-specific constraints on recombination. This work substantiated that recombination among betacoronaviruses is limited by the genome similarity between their parental viruses. We conclude that SARS-CoV-2 likely acquired unique features through recombination with closely related circulating sarbecoviruses (most likely from the same species) that co-existed geographically.

**IMPORTANCE:**

Understanding the evolutionary events that led to SARS-CoV-2 emergence, spillover, and spread is crucial to prevent, or at least be prepared for, the same type of occurrence in the future. Given that SARS-CoV-2 has some characteristics not found in other closely related viruses, we aimed to systematically assess how likely these unique features may have been acquired through recombination. We found that, although recombination is a frequent phenomenon among betacoronaviruses, it is mostly limited to closely related members of the same species. Therefore, we conclude that the most likely scenario involved feature acquisition from recombination with a closely related virus that was circulating in a geographically overlapping area or through a different biological process, but not recombination from a virus of a different species, genus, or subgenus.

## INTRODUCTION

Severe acute respiratory syndrome coronavirus 2 (SARS-CoV-2) is the causative agent of the coronavirus disease 2019 (COVID-19) pandemic, associated with ≈ 7.1 1million worldwide deaths (1) since those first reported in December 2019. SARS-CoV-2 is a highly transmissible positive-sense RNA virus related to predominantly bat viruses assigned to family *Coronaviridae*’s genus *Betacoronavirus* (2). How SARS-CoV-2 evolved from its ancestors and adapted to infect humans is an area of active research.

Among the 15 officially classified viruses that comprise genus *Betacoronavirus* (2), only five are known to infect humans: human coronavirus HKU1 (HCoV_HKU1; subgenus *Embecovirus*), human coronavirus OC43 (HCoV_OC43; subgenus *Embecovirus*)⁠, Middle East respiratory syndrome coronavirus (MERS-CoV; subgenus *Merbecovirus*), and SARS-CoV and SARS-CoV-2 (both assigned to the same species in subgenus *Sarbecovirus*). Importantly, most of the betacoronavirus spillover events to the human population have occurred in the last 22 years (SARS-CoV, 2003; MERS-CoV, 2012; and SARS-CoV-2, 2019) (3, 4)⁠. HCoV_HKU1, which is related to rodent betacoronaviruses, was reported in 2004 (5) but had been circulating in humans for a while before identification. Given the frequency of these events, it is likely that many other unreported zoonotic sarbecovirus spillovers had occurred previously but did not result in effective human-to-human transmission.

Both the MERS-CoV and SARS-CoV genome sequences have signs of early human adaptation after their initial zoonotic spillover (6, 7). However, several analyses concluded that the immediate ancestor to SARS-CoV-2 would have been capable of transmission among humans prior to the first reported human cases in 2019 (8).

## RESULTS

### Identification of ancestral sarbecovirus strains

As a result of the COVID-19 pandemic, numerous novel coronavirids were discovered all over the world and the majority remain to be named and classified. For instance, subgenus *Sarbecovirus* currently only harbors SARS-CoV and SARS-CoV-2 officially in a single species (*Betacoronavirus pandemicum*) (2), but numerous other viruses have been grouped with these viruses. Here, we will refer to these unclassified viruses as “sarbecovirus strains.”

Sarbecovirus strain RaTG13, isolated from an intermediate horseshoe bat (*Rhinolophus affinis* Horsfield, 1823) in Yunnan Province, China, in 2013, was identified early in the COVID-19 pandemic as the most-closely related ancestor to SARS-CoV-2, sharing more than 95% genome sequence similarity (9, 10)⁠. Subsequently, other closely related sarbecovirus strains, including RmYN02, isolated from a Malayan horseshoe bat (*Rhinolophus malayanus* Bonhote, 1903), and two strains sampled in China's Guangxi Province and Guangdong Province, respectively, that were reported to be genomically highly similar to SARS-CoV-2 (9–12). More recently, additional related viruses have been detected in bats in Laos (13)⁠. The receptor-binding domains (RBDs) of the spike (S) proteins of these strains bind efficiently to human angiotensin converting enzyme 2 (ACE2), the cell-surface receptor of SARS-CoV and SARS-CoV-2. According to the intraspecies distance threshold determined by the International Committee on Taxonomy of Viruses (ICTV) (2), all of these strains are members of species *Betacoronavirus pandemicum* (11).

### Unique features of SARS-CoV-2

Compared with SARS-CoV and all sarbecovirus strains of the species, SARS-CoV-2 possesses unique characteristics, such as a polybasic furin cleavage site in the S protein that has been associated with increased virulence and transmission (14)⁠. Importantly, putative furin cleavage sites are present in the S proteins of some non-sarbecovirus betacoronaviruses (e.g., MERS-CoV) (15)⁠. It has been postulated that the absence of a furin cleavage site in non-SARS-CoV-2 sarbecoviruses is due to the route of transmission in their host reservoirs; free-tailed bats (*Chaerephon/Mops* spp.) and horseshoe bats (*Rhinolophus* spp.) transmit viruses through the fecal–oral route, for which uncleaved spikes appear beneficial (16).

The pathogenic potential of betacoronaviruses is determined by two separate components: (1) acquisition of an RBD that might drive zoonotic spillover to humans (i.e., an RBD that can interact with a human receptor) and (2) efficient processing of the S protein that facilitates respiratory person-to-person transmission (17, 18). Several genetic mechanisms have been suggested as ways of gaining these capabilities: mutational drift (19), polymerase slippage (20), and virus recombination. Polymerase slippage is unlikely to generate the furin cleavage site in the S protein on the basis of the observed sequence motifs in the closest ancestor and the SARS-CoV-2 Wuhan-1 strain, but recombination is an extremely common phenomenon among coronavirids and plays a significant role in their evolution, with the S protein, including its RBD, identified as a recombination hotspot (21–24)⁠. Recombination has been linked to the emergence of new coronavirids, such as SARS-CoV (25), and the evolution of new variants of SARS-CoV-2 (26)⁠. Initial hypotheses suggested that SARS-CoV-2 acquired its RBD through recombination with a sarbecovirus found in pangolins (27)⁠. However, subsequent research disputed this claim, proposing alternative scenarios involving more ancestral recombination events with other closely related sarbecoviruses, such as SARS-CoV, or sarbecovirus strains, such as RaTG13 (17, 18, 23, 24)⁠.

### Recombination analysis

The SARS-CoV-2-unique S protein furin site among sarbecoviruses suggests that, if recombination was responsible for its acquisition, it likely involved a genetically distant parental virus. To assess the possibility of distant recombination events, we conducted a comprehensive recombination analysis study that assessed the likelihood of genetic exchange among coronavirids across various taxonomic ranks and genetic distances. This probabilistic approach aimed to infer the potential for genetic material exchange among coronavirids, irrespective of the detection of specific recombination events or the availability of a specific parental virus. By investigating the genetic distance among potential parental viruses at different taxonomic ranks (across vs within genera, subgenera, and species), we hypothesized that these analyses would provide insight into the likelihood of SARS-CoV-2 acquiring unique features, including the furin cleavage site, through recombination with a distantly related virus.

### Recombination patterns among coronavirids: insight from comprehensive analyses

#### Recombination occurs more frequently among closely related viruses

We conducted a comprehensive set of recombination tests using the RDP4 software package to identify and quantify recombination events among coronavirids. Our analysis included a data set of 206 genome sequences representing all established betacoronavirus species (2).

**Fig 1:**
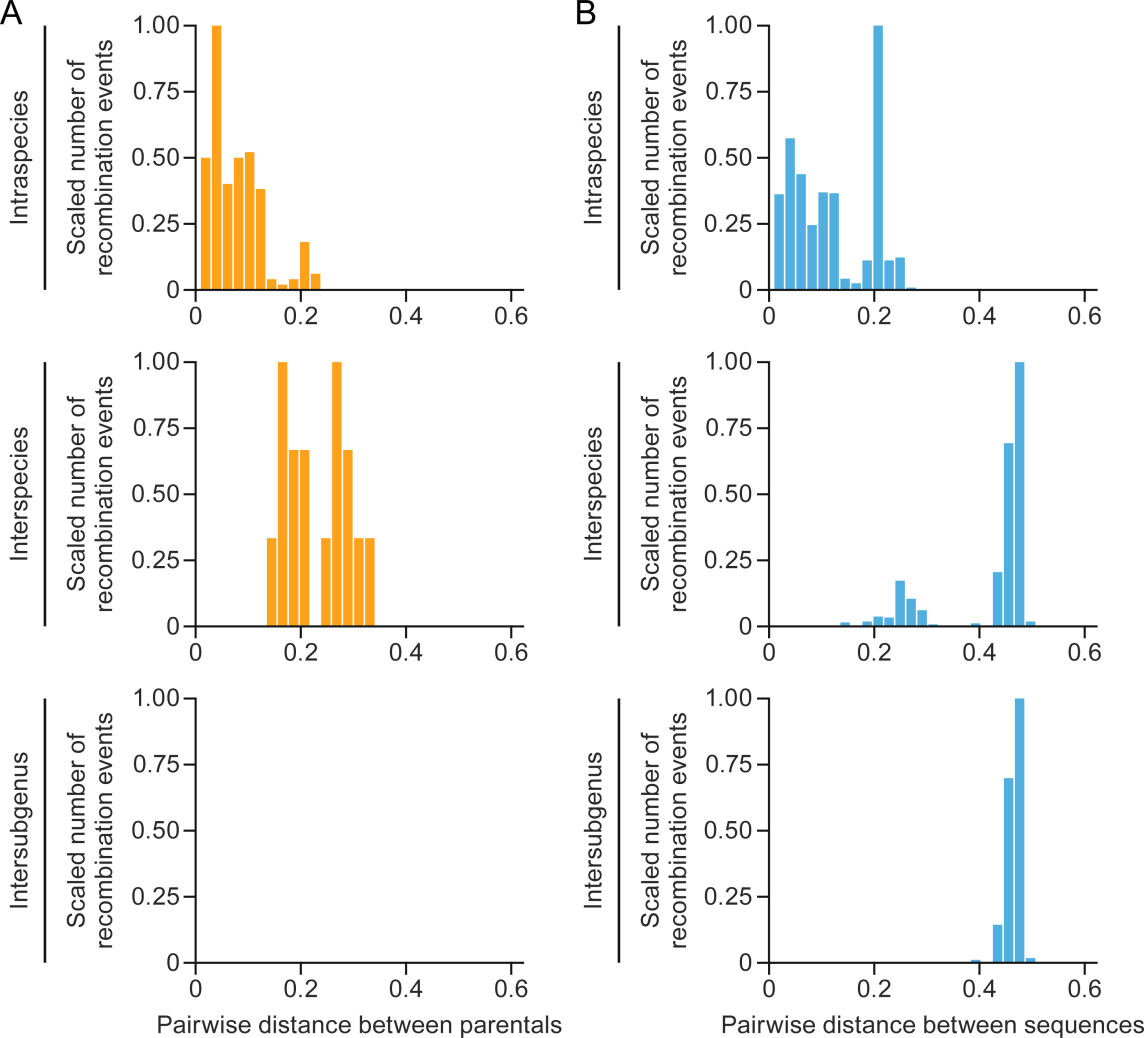
Recombination among betacoronaviruses is biased toward lower pairwise genetic distances. Splitting at different taxonomic ranks (intraspecies, interspecies, and intersubgenus): (**A**) the distribution of recombination events involving parental viruses; (**B**) the distribution of all distances among sequences in the whole data set. The number of recombination events is scaled based on the highest value (which get the value of 1).

Considering the whole alignment, the average intraspecies distance was 0.11, with a standard deviation (sd) of 0.07. Average interspecies distance was 0.42 (sd = 0.09) (Fig. 1B).

We identified a total of 386 potential recombination events, of which 187 were eliminated due to obvious alignment errors, having support from fewer than three tests in the RDP package, lack of phylogenetic informativeness, and/or the absence of distinction of the recombinant segment’s phylogeny from the segment without any recombination signal. Among the 199 validated events, 183 (92% of the total) occurred among parental virus strains from the same species, whereas only 16 events (8%) involved those from different species. The average distance between parents from intraspecies recombination events was 0.08 (sd = 0.05). The average distance between parents from interspecies recombination events was 0.23 (sd = 0.06) (Fig. 1A).

Importantly, no recombination events between parental viruses of different subgenera were detected (Fig. 1).

Next, we performed a similar analysis on a data set of reference sequences representative of the four established coronavirid genera in subfamily *Orthocoronavirinae* (i.e., *Alphacoronavirus*, *Betacoronavirus*, *Gammacoronavirus*, and *Deltacoronavirus*). We detected a total of 72 events. Notably, only one intergenus event was validated, specifically between alphacoronaviruses and deltacoronaviruses (Fig. S1 and S2).

An analysis of the distribution of pairwise genetic distances for these events revealed a distinct bias. The distribution showed an enrichment of recombination events among closely related sequences. This bias was clearly observed when the distribution was stratified into intraspecies, interspecies, and intersubgenus events (Fig. 1). Remarkably, approximately 90% of the recombination events occurred among parental virus strains with genetic distances of less than 20%, a threshold rarely surpassed among individual virus strains of the same species.

Together, these findings provide valuable insight regarding the patterns of recombination among coronavirids, highlighting the preference for closely related virus strains as major contributors to recombination events.

#### The ancestry context of recombination events is limited

**Fig 2:**
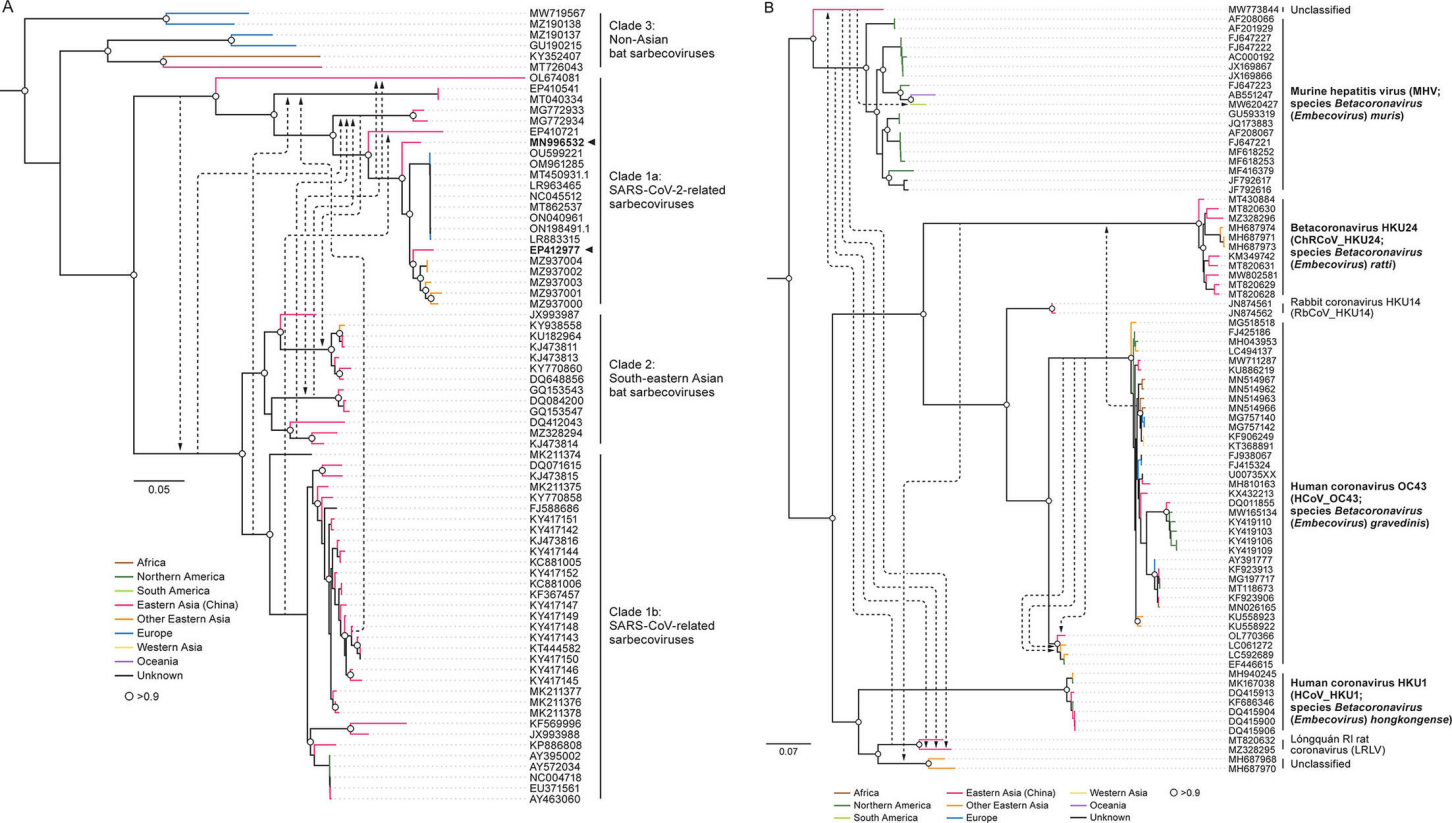
The ancestry of recombination events in genus *Betacoronavirus*. (**A**) Interclade recombination events in *Sarbecovirus*, the subgenus with the highest number of events. Sarbecoviruses are commonly divided into clades, thus sequences (identified via GenBank accession numbers) are classified accordingly. (**B**) Interspecies recombination events in *Embecovirus*, the subgenus with the second-highest number of events. Each arrow represents a recombination event. Arrows in both subfigures represent individual recombination events. The base of an arrow is at the minor parental ancestor (donor of recombination fragment), and the head of the arrow points to the ancestor (or sequence) considered for recombination. The names and abbreviations of officially classified viruses are emphasized in bold print (2). Trees were midpoint-rooted, and directionality of recombination (arrows) is given from the results of the receptor-binding domain (which specifies recombinants and parents). White circles represent nodes with support values > 0.90.

By analyzing *Orthocoronavirinae* phylogenies and mapping the interspecies events, we observed that the ancestry of recombination events is typically limited, affecting either terminal branches or internal branches within subclades of a single species (Fig. 2; Fig. S4). Although SARS-CoV and SARS-CoV-2 are currently not assigned to separate species by the ICTV (2, 11), we distinguished them in our analyses to investigate gene flow to and from the SARS-CoV-2 clade. This distinction was prompted by the significant impact of recombination within subgenus *Sarbecovirus*, driven by the ongoing COVID-19 pandemic. Our analysis concluded that, as expected, recombination events primarily occur among sarbecovirus strains within the same species, particularly among sequences closely related to SARS-CoV and SARS-CoV-2 (Fig. 2A). Notably, these recombination events predominantly involve virus strains sampled from hosts with a geographic overlap (Fig. 2A) (28).

#### Differential distribution of recombination events in different genome regions of viral species/hosts

In addition to investigating the frequency of recombination events across genetic scales, we examined their distribution across the viral genome, focusing on potential variations between intraspecies and interspecies events. Our analysis revealed distinct patterns of the impacted genome regions, specifically region-specific constraints on interspecies and intraspecies recombination.

**Fig 3:**
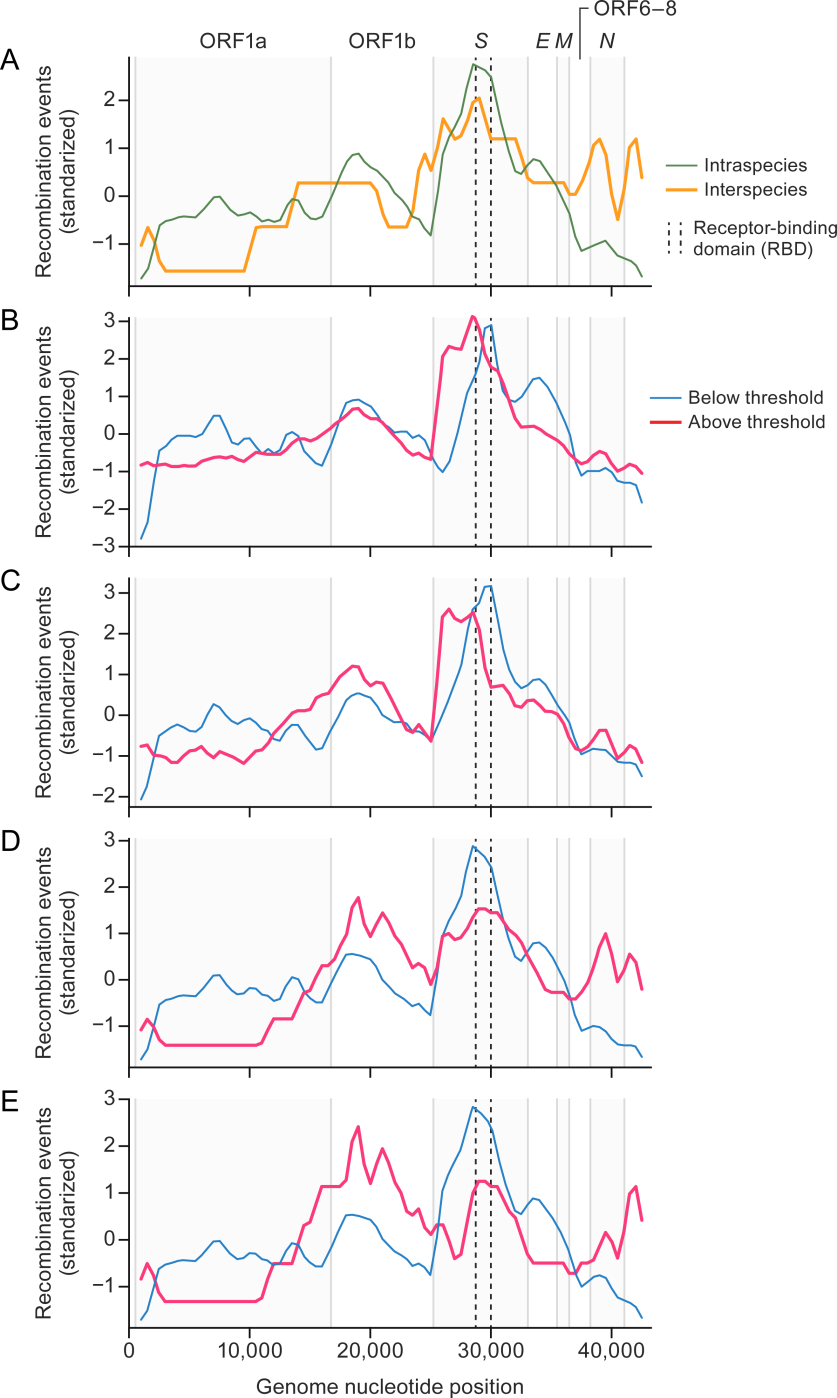
Interspecies and intraspecies recombination events tend to occur at different genome locations in betacoronaviruses. (A) Sliding window analysis (length 1,000 nt, steps = 500 nt) of the mean number of recombination events along the genome. Because the number of events is close to zero, expressed in log1p scale: intraspecies (thin green line) vs interspecies (thick orange line). (B–E) Sliding window analyses, splitting the groups by pairwise genetic distances (0.08, 0.10, 0.15, and 0.20) between parental viruses: events below the threshold (thin blue line) vs events above the threshold (thick red line). The distribution of recombination events is shown transformed by standardization (mean = 0, SD = 1). Vertical lines represent open reading frame (ORF) boundaries; dashed lines show the receptor binding domain within the spike [S] protein ORF.

Overall, the region responsible for encoding the S protein emerged as the most affected by recombination (Fig. 3). Conversely, the ORF1 region, particularly ORF1a, exhibited the lowest frequency of recombination events. It is noteworthy that the recombination-free concatenated alignment primarily consists of segments derived from ORF1 (Fig. S3).

The distribution of recombination events along the betacoronavirus genome was significantly different between intraspecies and interspecies events (Kolmogorov–Smirnov test: distance (*D*) = 1, *P*-value < 0.001). Events involving sequences assigned to different betacoronavirus species were less prone to affect regions of ORF1a and ORF1b (Fig. 3A). Given that the number of interspecies recombination events was low, we repeated this comparison at different thresholds of pairwise genetic distance among parental viruses. In a similar way to the intraspecies vs interspecies comparison, sensitivity analyses revealed that such differences were consistent in all comparisons (Kolmogorov–Smirnov tests: *D* > 0.50, *P*-value < 0.001). They all reflected a lower number of recombination events in ORF1a, ORF1b, and the *S* ORF in the group of events from more-distantly related parental viruses, as well as an increase in the 3′ end (ORF6–ORF8 and the *N* ORF) that could be observed at higher thresholds (events derived from more-distantly related parental viruses) (Fig. 3B through E). These findings underscore the existence of region-specific constraints on recombination among betacoronaviruses, highlighting the reduced occurrence within ORF1a, ORF1b, and the *S* ORF, which suggests that they play an important role in host adaptation and potential functional implications that warrant further investigation.

### Constraints on recombination events among coronavirids: implications for SARS-CoV-2 adaptation

We also explored the potential of sarbecoviruses to recombine with distantly related viruses. Specifically, we quantified the frequency of recombination across a wide range of genetic differences and taxa. Unlike previous reports that have tested recombination at the subgenus rank (21, 23, 24, 29)⁠, our study included representatives of all *Betacoronavirus* subgenera and species.

## DISCUSSION

Our findings demonstrate that recombination predominantly occurs among closely related virus strains, almost exclusively those assigned to the same virus species. Notably, the frequency of recombination sharply declines among strains with pairwise genetic distances exceeding 0.20. Consequently, recombination events among viruses belonging to different species are rare, and no evidence of recombination events among sequences of betacoronaviruses of different subgenera were identified. These results align with previous studies on alphaherpesviruses (family *Herpesviridae*) and lentiviruses (family *Retroviridae*), which are characterized by recombination events being mainly limited to closely related strains (30–32).

Our observations suggest the existence of barriers that impede the recombination of distantly related genomes. These barriers might be associated with a lack of a single host cell that parental viruses can co-infect, lack of overlap of replication sites, difficulty in generation of replication-competent chimeric viruses, or different geographic locations, among others. Consequently, the formation of mosaic genomes resulting from regions of distantly related ancestors is significantly restricted. Our findings emphasize the low probability of gain of function through recombination of coronavirids from different species and the even lower likelihood of feature transfer across subgenera. Hence, genetic relatedness emerges as a critical factor limiting the occurrence of virus recombination that should be taken into consideration alongside other barriers (e.g., geographic overlap of parental viruses and ability to infect the same host or cell type) (29).

We identified only a single reliable case of intergenus recombination. This ancestral event affected the S protein, likely resulting from the recombination of the *S* genes of an alphacoronavirus and a deltacoronavirus. This recombination event has been reported before, as a phylogenetic incongruence between alpha- and betacoronaviruses (33).

Our comprehensive inclusion of sequences from all four *Orthocoronavirinae* genera enabled a better characterization of this recombination event. These results show that, although the probability of recombination sharply decreases with genetic distance, its occurrence is still possible and may have relevant effects on coronavirid evolution. Indeed, other cases of recombination of distantly related viruses have been reported. For instance, the discovery of Rousettus bat coronavirus GCCDC1 (Ro-BatCoV_GCCDC1) has been linked to the occurrence of heterologous recombination between a betacoronavirus/nobecovirus and an orthoreovirus (family *Reoviridae*) (34).

The area surrounding the S protein has been recognized as a recombination hotspot associated with coronavirus adaptation to new hosts (23, 24, 29, 35), but our analysis indicates that ORF6–ORF8 and the *N* ORF also have a higher propensity for interspecies recombination than the *S* ORF, a consistent result even when different thresholds for pairwise genetic distance among parental viruses were applied. These findings suggest the involvement of these proteins in the adaptation process. Notably, the expression products ORF6–ORF8 and the *N* ORF are known to engage in various interactions with the host, such as suppressing interferon responses and inducing cell cycle arrest (17, 18, 36, 37). Sarbecoviruses differ in their engagement with the interferon systems of their hosts, highlighting the potential functional implications of recombination events among these ORFs (37).

Conversely, ORF1a was associated with a lower frequency of recombination at different thresholds. Overall, this region represented a “cold spot” of recombination. In fact, ORF1 is the only area mostly included in the “low-recombination” concatenate used to verify the detected recombination events. ORF1 interspecies recombination events were significantly decreased with intraspecies cases. This observation still held true after repeating the analysis by comparing recombination events derived from the top 50% of most closely related parental viruses with those from the top 50% of more distantly related ones. This suggests that ORF1 is selectively constrained with respect to recombination activity compared to other genome regions.

Our results indicate that, even in cases of interspecies recombination, most events involve closely related virus strains, thereby potentially exerting a limited impact on the evolution of a virus. Given the special interest in SARS-CoV-2, we focused on the recombination trends among sarbecoviruses, differentiating SARS-CoV and SARS-CoV-2 despite their being members of the same species. Recombination in sarbecoviruses is limited geographically, with Asian clades (which include SARS-CoV, SARS-CoV-2, and their most closely related sarbecovirus strains) associated with many recombination events but complete absence of recombination among virus strains of non-Asian origin. Interestingly, within the Asian sarbecovirus clades, recombination among the subclade that includes SARS-CoV-2 occurs with viruses and strains of the other two Asian subclades (SARS-CoV and closely related strains from South-eastern Asia), in which we detected a frequent flow of fragments through recombination from SARS-CoV and its closest relatives to SARS-CoV-2 lineages. In total, we detected eight recombination events shaping the genome of the SARS-CoV-2 subclade from donors belonging to different groups in a time span of ≈800 years (time to the most recent common ancestor between SARS-CoV and SARS-CoV-2) (21)⁠.

Although occurring less frequently, it is important to highlight a few ancestral recombination events in the other direction (from an ancestor of SARS-CoV-2 to an ancestor of SARS-CoV). One of these is an ancestral event in which the most recent common ancestor of SARS-CoV and its closest bat relatives from Southern Asia would have acquired a fragment that spans regions encoding ORF6–ORF8, matrix protein (*M*) ORF, and the *N* ORF from a common ancestor of SARS-CoV-2 and pangolin viruses from China's Guangxi Zhuang Autonomous Region. These results exemplify a sarbecovirus evolution scenario that involves exclusive recombination among members of this subgenus.

### Conclusion

Altogether, we have found that, although recombination is a frequent phenomenon among betacoronaviruses, it is limited by genomic similarity. Host geographic range physically limits recombination (29)⁠, and viral genomic similarity can limit recombination by reducing recombination rates across dissimilar sequences and generation of viable mosaics among distant genomes. Thus, from our probabilistic analyses of recombination, we can conclude that the most likely scenario in which SARS-CoV-2 would have acquired some of its unique features, such as the S protein furin cleavage site, is a recombination event among sarbecovirus strains co-existing geographically and most likely belonging to the same species.

## MATERIALS AND METHODS

### Sample collection: coronavirid genome sequences

A total of 1,473 betacoronavirus genome sequences were obtained from the National Center for Biotechnology Information (NCBI) in February 2022. To ensure the inclusion of only relevant sequences, exclusion criteria were applied using specific keywords, such as “not listed,” “patent,” "clone," "construct," "provirus," "proviral", "plasmid," "chimera," "chimeric," "cell culture," "replicon," "vector," and "unverified".

Considering the substantial number of SARS-CoV-2 sequences available, a curated data set containing 1,694 genome sequences of SARS-CoV-2 was downloaded from NextStrain (https://nextstrain.org/SARS-CoV-2/#datasets) in May 2022 (38). To eliminate redundancy, a clustering analysis was conducted using uclust, setting a threshold of 99% sequence similarity. Subsequently, only one representative sequence from each cluster was retained in the final data set (39)⁠. This data set, comprised of 206 sequences, provided a representative sample encompassing all five subgenera (*Embecovirus*, *Hibecovirus*, *Merbecovirus*, *Nobecovirus*, and *Sarbecovirus*) and their, in total, 14 species for 15 classified betacoronaviruses (2).

Additionally, to facilitate intergenus recombination analyses, a set of 66 reference sequences representing all established species in the *Coronaviridae* family was also downloaded from NCBI. Sequence alignments were performed using MAFFT7 with the “FFT-NS-i” strategy, which uses an iterative refinement method (40)⁠.

### Recombination detection and analysis

To identify potential recombination events, we used seven recombination detection methods available in the RDP4 software package (Geneconv, Bootscan, Maxchi, Chimaera, SiScan, 3seq) (41)⁠. Default parameters were used for these analyses, with a significance threshold set at *P*-value = 0.05, Bonferroni-corrected. The recombination analyses were conducted on two distinct data sets: (i) an alignment of betacoronaviruses and (ii) a data set representing the four *Orthornavirinae* genera (*Alphacoronavirus*, *Betacoronavirus*, *Gammacoronavirus*, and *Deltacoronavirus*).

### Validation procedures

To ensure the robustness and accuracy of identified recombination events, we implemented the following validation steps:

#### Consistency across multiple tests

Recombination events were considered valid when they were supported by at least three independent tests.

#### Phylogenetic information

The identified recombinant regions were assessed for their phylogenetic informativeness. This was achieved through quartet analysis using TREE-PUZZLE software (42)⁠.

#### Distinct tree topology

Recombination generates mosaics, in which the recombinant region should have a different evolutionary history than the rest of the genome. We compared the tree topology obtained from the recombinant regions with that derived from a concatenated alignment of genome regions having the lowest number of initial recombination events. These regions were found by counting the number of recombination breakpoints in a sliding window analysis (length = 1,000 nt, steps = 500 nt). The “recombination-free” concatenate was built from windows representing up to percentile 10 in the distribution of recombination breakpoints per window along the genome (Fig. S3). Maximum-likelihood phylogenetic inference was performed using PhyML (43)⁠, employing a GTR + GAMMA (4 cat) substitution model. Comparison of the trees was conducted using TREE-PUZZLE, with the expected likelihood weight and Shimodaira–Hasegawa tests.

#### Alignment quality and error assessment

Recombinant regions were scrutinized for potential alignment errors or low-quality signals that could affect the reliability of the analysis.

### Statistical analysis of recombination patterns among coronavirids

After the identification and validation tests, we explored the factors influencing recombination dynamics among coronavirids by obtaining the following information for each recombination event:

#### Maximum pairwise genetic distance

We calculated the maximum pairwise genetic distance between parental virus sequences using the Analysis of Phylogenetics and Evolution (ape) (44) and phytools (45) packages in R. This information enabled us to examine the distribution of recombination events across different genetic distances.

#### Genome coordinates of recombinant fragments

We recorded the precise genome coordinates of the recombinant fragments to identify specific regions and ORFs that were more susceptible to recombination.

#### Taxonomic information

We collected data on the species, genera, and order (taxonomy rank) of the parental viruses involved in recombination events. Additionally, we noted the countries from where the sequences were obtained. This information enabled us to compare the frequency of recombination events occurring within the same group (intragroup) vs across different groups (intergroup).

#### Distribution of recombination events

To investigate the distribution of events along the coronavirid genome involving sequences from viruses of different species, we quantified the number of occurrences along the genome through a sliding window of length of 1,000 nt moving at steps of 500 nt. The distribution of intraspecies vs interspecies events along the genome was assessed by means of Kolmogorov–Smirnov tests. Given that the number of interspecies events was very low, we repeated these analyses by splitting the whole set of recombination events into two groups based on different median pairwise genetic distance between parental viruses. We performed this analysis using four pairwise genetic distance thresholds: 0.08, 0.10, 0.15, and 0.20.

## Data Availability

The viral sequence data generated and analyzed in this study are publicly available through the GISAID and GenBank databases. The supplemental material includes the acknowledgment section for the sequences obtained in GISAID (https://www.gisaid.org) following the database’s access and usage guidelines. Specific accession numbers for sequences used in this study from GenBank are also provided in the supplemental material and are accessible through the National Center for Biotechnology Information (NCBI) database at https://www.ncbi.nlm.nih.gov/genbank.

## SUPPLEMENTAL MATERIAL

Fig. S1 jvi.01100-24-s0001.tif The case of intergenus recombination among deltacoronaviruses and alphacoronaviruses.

Fig. S2 jvi.01100-24-s0002.tif Phylogeny based on recombinant region among deltacoronaviruses and alphacoronaviruses.

Fig. S3 jvi.01100-24-s0003.tif Distribution of recombination events along the betacoronavirus genome alignment.

Fig. S4 jvi.01100-24-s0004.tif Interspecies recombination events among (A) merbecoviruses and (B) nobecoviruses.

Supplemental legends jvi.01100-24-s0005.docx Legends for Fig. S1 to S4.

Table S1 jvi.01100-24-s0006.xls Genbank and GISAID accession numbers for sequences used in this study.

ASM does not own the copyrights to Supplemental Material that may be linked to, or accessed through, an article. The authors have granted ASM a non-exclusive, world-wide license to publish the Supplemental Material files. Please contact the corresponding author directly for reuse.
