# Supplementary figures and images for: Recombination across distant coronavirid species and genera is a rare event with distinct genomic features
Source: J Virol. 2024 Nov 19;98(12):e01100-24. doi: 10.1128/jvi.01100-24 (PMC11650996; doi:10.1128/jvi.01100-24)

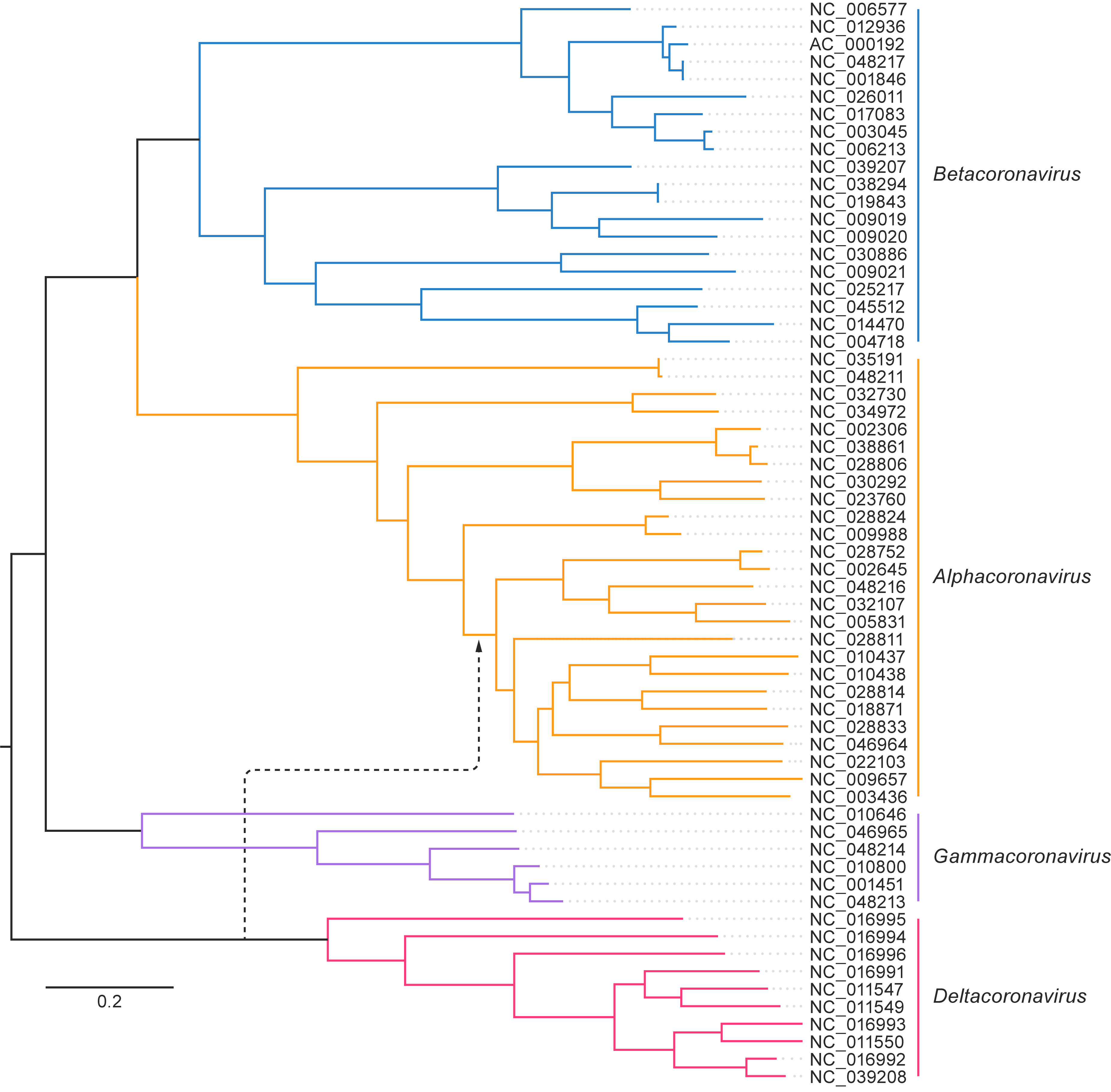

Supplement: Fig. S1 — The case of intergenus recombination among deltacoronaviruses and alphacoronaviruses. [file jvi.01100-24-s0001.tif]

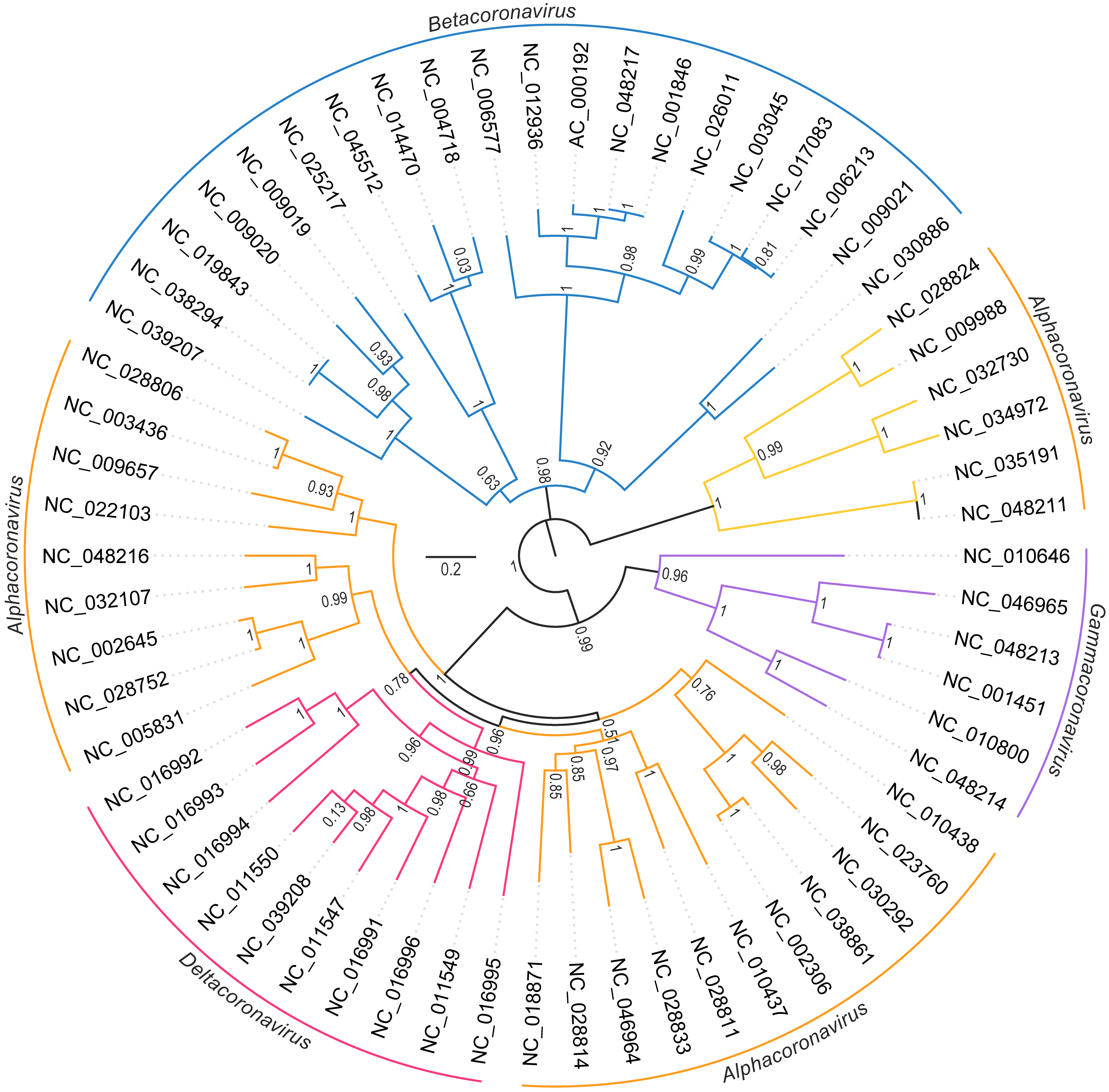

Supplement: Fig. S2 — Phylogeny based on recombinant region among deltacoronaviruses and alphacoronaviruses. [file jvi.01100-24-s0002.tif]

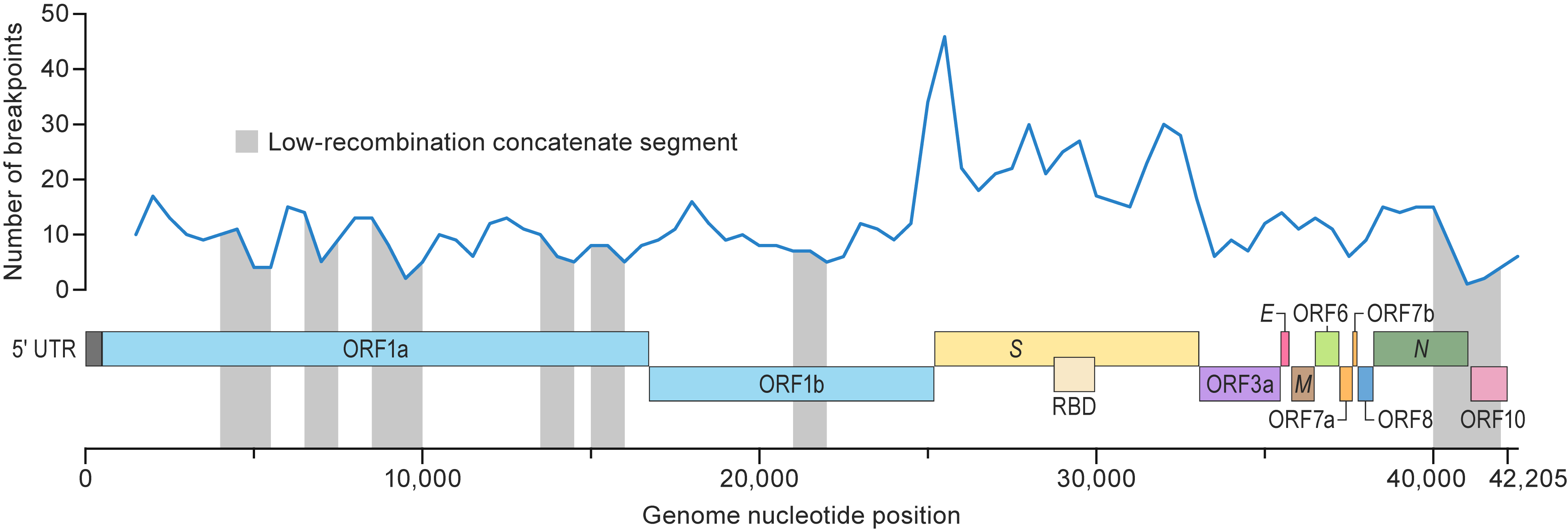

Supplement: Fig. S3 — Distribution of recombination events along the betacoronavirus genome alignment. [file jvi.01100-24-s0003.tif]

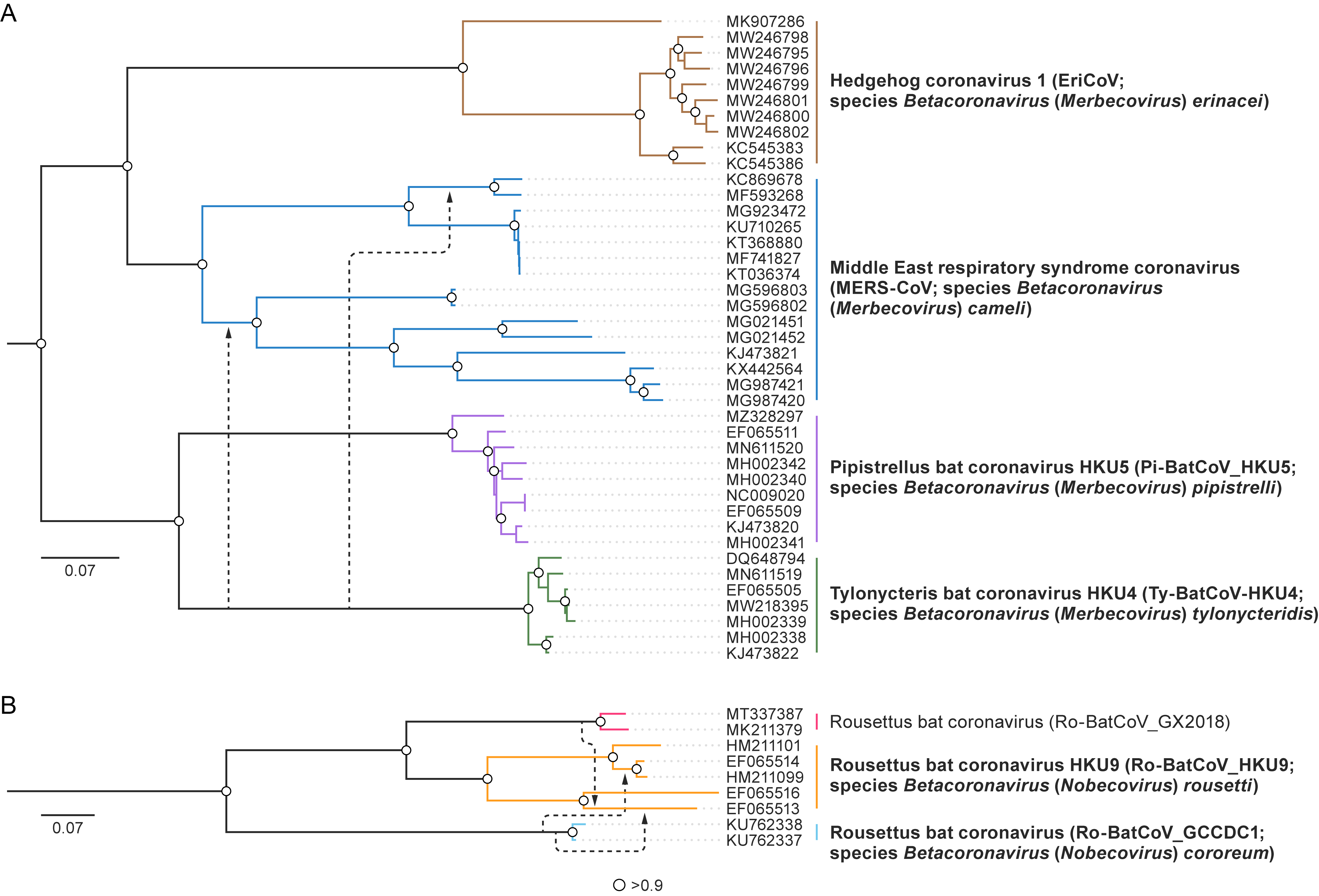

Supplement: Fig. S4 — Interspecies recombination events among (A) merbecoviruses and (B) nobecoviruses. [file jvi.01100-24-s0004.tif]
