# Supplementary material for: Recombination across distant coronavirid species and genera is a rare event with distinct genomic features
Source: J Virol. 2024 Nov 19;98(12):e01100-24. doi: 10.1128/jvi.01100-24 (PMC11650996; doi:10.1128/jvi.01100-24)
Supplement: Supplemental legends — Legends for Fig. S1 to S4. [file jvi.01100-24-s0005.docx]

**SUPPLEMENTAL FIGURE LEGENDS**

**Fig. S1 The case of intergenus recombination among deltacoronaviruses and alphacoronaviruses (nucleotide positions 21,962–24,270 [within the *S* open reading frame]).** Sequences are identified via RefSeq accession numbers. The arrow represents the recombination event. The base of the arrow is at the minor parental ancestor, and the head of the arrow points to the ancestor (or sequence) considered for recombination. Directionality of recombination (arrows) is given from the results of the receptor binding domain (which specifies recombinants and parents).

**Fig. S2 Phylogeny based on recombinant region (nucleotide positions 21,962–24,270 [within the *S* open reading frame]) among deltacoronaviruses and alphacoronaviruses.** Sequences are identified via RefSeq accession numbers.

**Fig. S3 Distribution of recombination events along the betacoronavirus genome alignment (RefSeq accession number NC_045512 Wuhan-1 reference coordinates).** Grey columns represent the regions included in the low-recombination concatenate segment. UTR, untranslated region; ORF, open reading frame; *S*, spike protein ORF; RBD, receptor binding domain; *E*, envelope protein ORF; *M*, matrix protein ORF; *N*, nucleocapsid protein ORF. ORFs are mapped according to their position in the alignment, based on RefSeq accession number NC_045512 Wuhan-1 reference. Note that positions reflect nucleotide positions in the alignment. The jagged blue line graphs the number of recombination breakpoints identified in each region.

**Fig. S4 Interspecies recombination events among (A) merbecoviruses and (B) nobecoviruses.** Each arrow represents a recombination event. Each arrow represents a recombination event. Arrows in both subfigures represent individual recombination events. The base of an arrow is at the minor parental ancestor (donor of recombination fragment), and the head of the arrow points to the ancestor (or sequence) considered for recombination. The names and abbreviations of officially classified viruses are emphasized in bold print ([2](#_ENREF_2)). Trees were midpoint-rooted. Trees were midpoint-rooted, and directionality of recombination (arrows) is given from the results of the receptor binding domain (which specifies recombinants and parents). White circles repre
